# From crisis to care: exploring the resilience of pediatric urologists in tackling complex urological challenges in a resource-limited country during volunteer campaigns: a qualitative study

**DOI:** 10.3389/fpubh.2025.1486283

**Published:** 2025-04-17

**Authors:** Anas Aboalsamh, Yousef Bassi, Dana Khafagi, Sarah Ali AlShamrani, Ahmad A. AlZahrani, Abdullah Mesawa, Basim Alsaywid

**Affiliations:** ^1^Faculty of Medicine, King Abdulaziz University, Jeddah, Saudi Arabia; ^2^Education and Research Skills Directory, Saudi National Institute of Health, Riyadh, Saudi Arabia; ^3^Urology Section, Department of Surgery, King Abdulaziz Medical City, Ministry of National Guard, Jeddah, Saudi Arabia; ^4^Urology Department, Dr. Soliman Fakeeh Hospital, Riyadh, Saudi Arabia

**Keywords:** pediatric urology, resource-limited settings, humanitarian surgery, complex urological disorders, global health, healthcare access

## Abstract

Surgeons operating in resource-limited settings encounter unique challenges due to the scarcity of materials and resources. Complex urological disorders (CUDs) such as bladder exstrophy, cloacal exstrophy, and posterior urethral valves, prevalent in these settings, often lead to varying surgical outcomes. This study aims to understand the experiences of surgeons treating pediatric patients with varying CUDs in a setting where resources are scarce through a qualitative phenomenological approach. We conducted six in-depth interviews with six pediatric urologists and surgeons who participated in humanitarian missions sponsored by the King Salman Humanitarian and Relief Center. The interviews, analyzed using the Nvivo v14.23.0 software, revealed common themes: inadequate equipment, lack of trained personnel, infrastructure challenges, emotional and physical tolls, and the need for effective communication and collaboration with local healthcare teams and providers. The findings highlighted the surgeons' adaptive strategies and resilience in overcoming these obstacles, emphasizing the critical role of support from humanitarian organizations. The study underscores the importance of ongoing education for local medical staff, the potential of telemedicine, and the need for consistent presence in resource-limited areas to improve patient care and outcomes. Addressing these challenges requires a concerted effort to optimize resources, enhance training, and support healthcare providers' wellbeing in these demanding environments.

## Introduction

Surgeons operating in resource-limited settings face a unique set of challenges that often require providing care beyond their usual training. These settings often lack materials and resources commonly available in more affluent environments (1). In Less economically developed countries (LEDCs), a higher prevalence of complex disorders is observed compared to More Economically Developed Countries (MEDCs), where early intervention is common (2). Among these complex disorders, a significant proportion falls under complex urological conditions (CUDs). The term CUDs is an umbrella term which encompasses congenital and acquired conditions covering both genetic or environmental origins of disease. Notable examples include bladder exstrophy, cloacal exstrophy, epispadias, proximal hypospadias, ambiguous genitalia, posterior urethral valves, prune belly syndrome, and multi-cystic dysplastic kidney. Varying surgical outcomes have been noted in resource-limited settings when looking at CUDs (3).

CUDs are primarily caused by mesoderm-derived birth defects during the 4^th^ week of gestation, when the urogenital system begins to separate. Any disruption or mutation in this process can lead to CUDs; environmental factors may also play a role (4). While numerous studies have documented treatment and management options for complex urological conditions in resource-rich settings usually in MEDC's (5–7) DDI, exploring innovative approaches in resource-limited settings becomes crucial.

The research on treatment and management modalities for complex urological conditions in resource-limited settings presents many diverse perspectives which help shed light on the challenges faced. One study highlights the utilization of the Mainz II pouch, an internal urinary diversion, for primary closure and diversion in patients ineligible for primary closure (8). Another study emphasizes the need for holistic care in Kenya, where challenges in performing osteotomy procedures may have contributed to less favorable post-surgical outcomes (9).

In Sudan, a well-designed paper reveals the striking reality that only 14 pediatric surgeons are available within the entire healthcare system as of 2020 (10). This is particularly alarming considering that more than 50% of the population is below the age of 14, and there is a significant prevalence of congenital anomalies (10). The burden falls on the shoulders of a limited healthcare workforce, leading to staff exhaustion and the depletion of medical resources.

A retrospective study spanning 5 years at the Ladoke Akintola University of Technology Teaching Hospital in Osogbo, Nigeria (July 2007–July 2012), examined cases of urethral stricture disease. The study found that the main reason for treatment delays was lack of funds (11). The study also pointed out that four out of every five patients were dependent, unemployed, or underemployed, and one out of every five went untreated due to financial constraints. Delays in evaluation were also observed due to the high patient load and limited facilities (11).

These challenges are amplified as congenital conditions tend to be more prevalent among individuals of low socioeconomic status, often due to the lack of prenatal care and higher exposure to teratogens (9). The information available on the management of complex pediatric urological diseases, within the context of limited supplies, staff, and funding, remains inadequate.

In the face of these challenges, surgical professionals continue to push boundaries finding new ways to provide quality care. Their resilience, dedication, and passion for providing humanitarian aid shine through, showcasing a new wave of surgical innovation. This innovation aims to bridge the gap and revolutionize care provided in resource limited settings through leveraging expertise, available resources, while also collaborating with organizations such as The King Salman Humanitarian and Relief Center.

Surgical professionals continuously strive to find innovative solutions while advocating to improve resources, inspiring a collective effort toward enhanced care for patients in resource-limited settings. By amplifying their voices, raising awareness, and driving collaborative initiatives, we can make a profound difference in the lives of those affected by the burden of complex urological conditions.

There is inadequate information describing management of complex pediatric urological diseases in resource limited settings and the burden on the physicians treating. The aim of this research project is to gain insights from surgeons operating on pediatric patients with complex urological conditions in resource-limited settings, with the goal of identifying the challenges they face while exploring innovative solutions that can enhance patient care and outcomes.

## Methodology

### Research design

Following IRB approval, we started a qualitative phenomenological study design to gather insight from surgeons who dealt with patients with complex urological conditions in resource-limited settings, specifically Yemen and various parts of Africa.

This specific design helps highlight both the obstacles encountered and the innovative strategies employed by the surgeons in order to holistically optimize patient care. Ultimately improving long-term surgical outcomes in these challenging environments.

#### Study setting

We conducted tele-interviews with different physicians over Zoom as it would be difficult to conduct in person interviews due to different geographical locations.

#### Study subjects

The inclusion criteria included pediatric urologists and surgeons, who performed complex urological surgical repairs during humanitarian missions. These missions were mainly organized by the King Salman Humanitarian and Relief Center between the years of 2018 to 2023. Eligible participants needed to have participated in at least one of these missions and have conducted pediatric surgeries within that mission.

#### Sample size and sampling technique

A purposive non-probability sampling technique was used to ensure selection of subjects with a diverse range of experience and direct field experience. This approach allowed the inclusion of professionals with varying case complexity exposure while ensuring a sufficient number of participants to achieve data saturation. We ended up interviewing 6 subjects which met the theoretical saturation.

#### Recruitment process

Potential participants were identified through networking doctors who had participated in previous humanitarian missions. Invitations to participate were sent either via email or by text message, the invitation explains the study's objectives and that participation is voluntary. Eventually participants were recruited based on availability and willingness to participate.

#### Saturation

Saturation was determined through a iterative process of data collection and analysis. Saturation was based on recurring patterns in responses and themes when it came to the participants experience. After 6 interviews no new concepts or differences emerged thus it was determined that the saturation had been reached.

#### Data collection

This study utilized a primary data collection method, interviews. The questions were presented to the study subjects during online Zoom meetings. Each Zoom meeting had no time limit to ensure participants would be able to express themselves freely. The research team was divided into two groups. Each group consists of two research team members designated the task of interviewers. Potential study subjects were assigned to one of the two groups. Each conducted interview consisted of four individuals: the study subject, two research members, and a dedicated witness who attended all interviews across all groups, to ensure the maintenance of quality. The interviews were recorded, and processed using the transcription website, “speak.ai,” then they were reviewed by the research team to ensure accuracy. Finally transcripts were imported into “nvivo 14,” a software used for comprehensive data analysis and interpretation.

**Figure 1:**
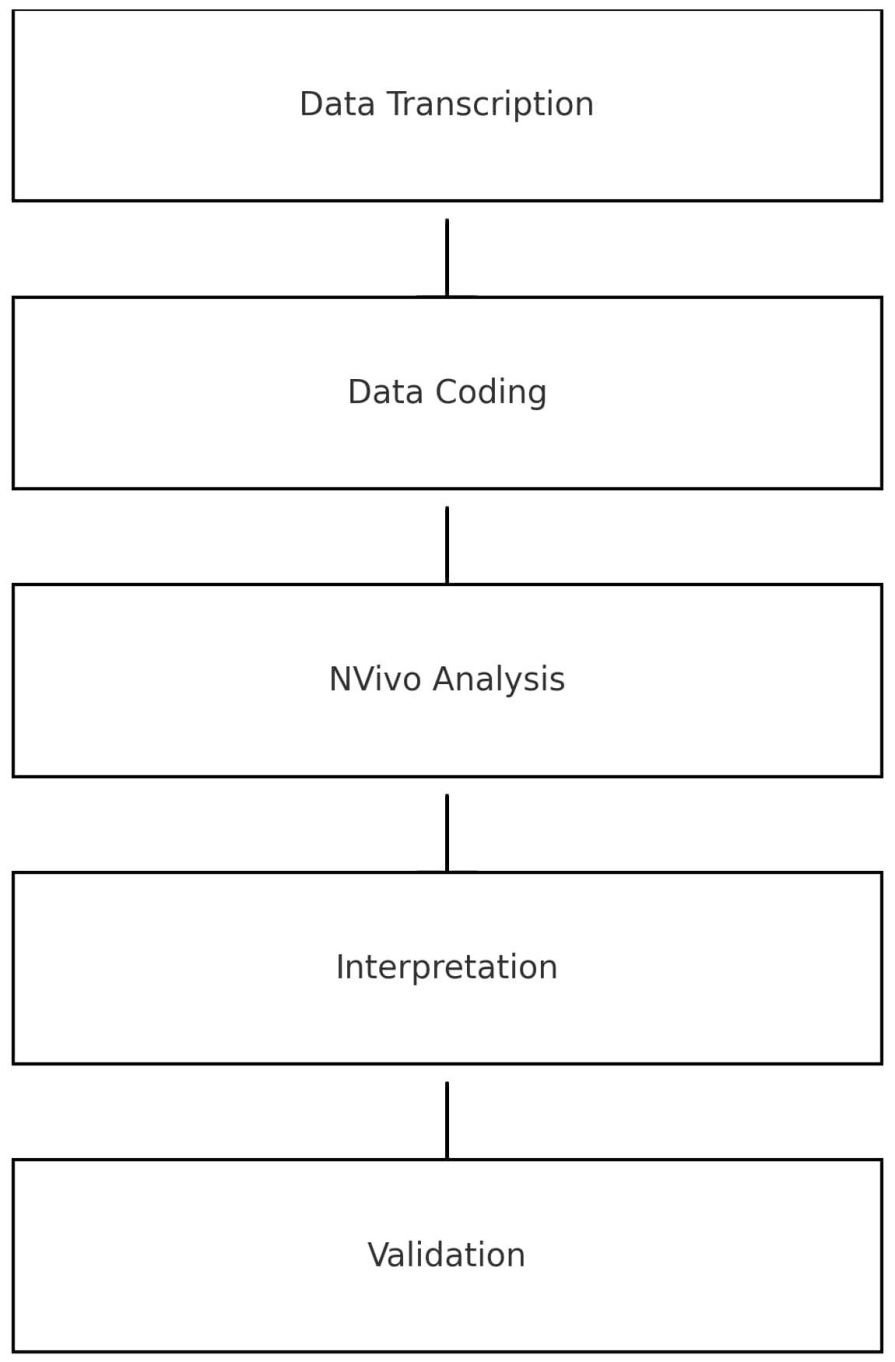
Displays the data analysis process in a linear flow chart manner.

Data analysis consisted of multiple stages including transcription, coding, Nvivo program analysis, interpretation and validation as highlighted in Figure 1.

#### Data transcription

The interviews were transcribed into written text format using a transcription service at speakai.co. Afterwards, the transcribed data is manually revised to ensure accuracy and facilitate easier analysis.

#### Data coding

In order to analyze the collected data, a meticulous coding process was performed. It was done by two members of the research team where they independently coded the same transcripts identifying significant segments or themes within the data and assigning appropriate labels. Then results are compared and discrepancies are discussed till a consensus is reached. Through an iterative approach, the codes were refined and grouped into broader categories, enhancing the overall understanding of the dataset.

#### Data NVivo analysis

We conducted an in-depth analysis using the NVivo program to examine the coded data. With the aim of identifying patterns, and contrasts across participants experiences. Thematic analysis was complemented by interpretive phenomenological analysis to uncover essential themes and interpret the underlying experiences.

#### Interpretation

The interpretation phase involved a thorough review of the coded data, where the research team reviewed the extracted key themes or patterns. This was done to ensure thorough unbiased NVivo analysis.

#### Validation

The validation process involved a meticulous verification of our findings, which included comparing them with existing literature, and subjected it to peer review by participants and within the research team to ensure credibility.

## Results

**Figure 2:**
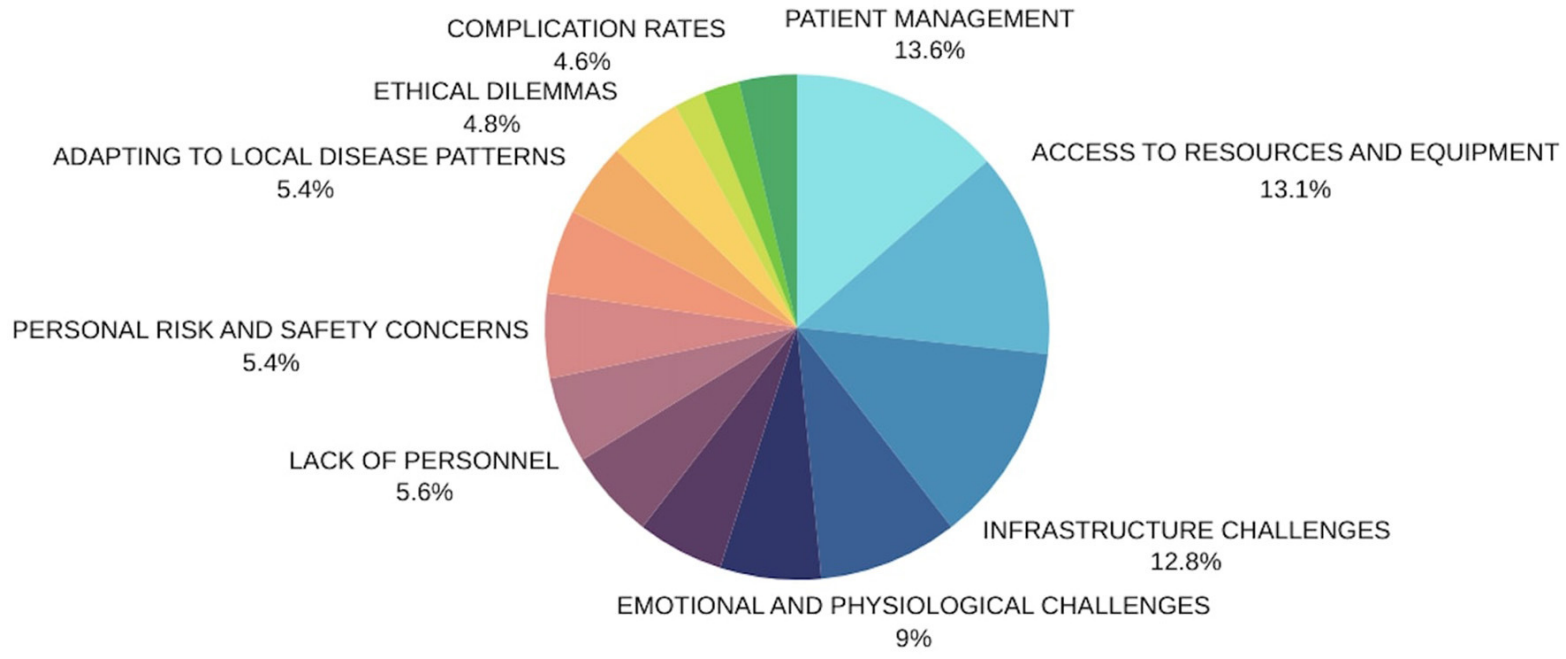
Presents the central themes and the frequency of each respective one throughout all interviews.

The aim of the study was to explore various surgeons' experiences during their visits to LEDCs. [Subject 1] interviewed for 62 min a consultant pediatric urologist whose been practicing for 6 years and volunteered on one humanitarian mission. [Subject 2] a urologist specialist who's been on three humanitarian missions and interviewed for 26 min. [Subject 3] a pediatric urologist consultant that's been practicing for 15 years interviewed for 38 min with one humanitarian mission. [Subject 4] a pediatric surgeon consultant who's been practicing for 23 years with more than fifty humanitarian missions to and was interviewed for 22 min. [Subject 5] a pediatric surgeon consultant that's been practicing for 8 years with five humanitarian missions, who's interview lasted 36 min. [Subject 6] a pediatric urologist consultant that's been practicing for 10 years with more than ten humanitarian missions and interviewed for 128 min. The data from each interview was stratified into codes in order organize central themes. The central themes are presented in Figure 2. Each code had specific characteristics into which appropriate quotations would fall. Quotes were selected by team members based on the impact they had during interviews, the aim was to present statements that were representative of the subjects experience.

### Patient management

The most frequently mentioned theme across the six interviews was “patient management,” mentioned 88 times. Participants discussed quality of care provided by doctors, noting both praise and critique, while also highlighting the rate of complications.

The first participant shared the struggles of prioritizing patient needs during appointments and the hurdles in obtaining necessary medications for post-surgery recovery. They expressed this with statements like, “For the poorer patients, getting the postoperative medicines was an enormous challenge,” and “It's overwhelming when people approach you in the hallways with X-rays and CT scans. It's heart-wrenching and makes you truly value our healthcare system.”

The second participant pointed out that in resource limited settings surgeries often took longer due to a lack of proper tools, saying, “The surgeries took a bit longer compared to what we're used to in Saudi Arabia because we just didn't have the right instruments for the procedures.”

Contrastingly, the third participant reported that despite patient management issues, their medical missions to resource limited settings were ultimately successful, with no major complications reported in the short or long term.

The fourth participant noted that the lack of treatment alternatives in resource limited settings forced them to proceed with surgery even when it wasn't the best option, confessing, “There were cases where we had to operate even though it wasn't the ideal treatment, but we had no other choices.”

Echoing the challenges mentioned by others, the fifth participant mentioned that patient care was complex, with significant recovery challenges leading to longer hospital stays due to the nature of surgical wounds.

The sixth participant emphasized the importance of having the right surgical tools, which were often scarce in resource limited settings, affecting patient care. They also mentioned making sure to bring essential supplies due to frequent shortages. Despite these challenges, they reported that surgical outcomes in Yemen were comparable to those in Saudi Arabia, crediting thorough preparation by aid organizations.

Each participant's experience underscores the complex reality of providing surgical care in resource limited settings and the resilience of medical teams in the face of resource limitations.

### Access to resources and equipment

The theme of “access to resources and equipment” is considered a critical theme in interviews, receiving 85 quotes, which underlies the significance of both quality and availability of resources for surgeons to provide care effectively.

One participant recalled the shocking scarcity of resources and equipment in less economically developed countries (LEDCs), often either facing delays or non-arrival of shipments, hindering the workflow for volunteers: “We were surprised that most of our equipment did not arrive,” and “in general, there is a lack, a huge lack of resources.” Another subject echoed this sentiment, detailing how volunteers had to get creative during surgeries to compensate for equipment shortages. However, it was noted that despite these challenges, the work outcomes were often exceptional, citing their resourceful combination of tools from different specialties to perform procedures successfully: “Actually, when we work with the pediatric groups, we need a small instrument. This is what we faced with. They don't, we don't find that instrument for working during a surgical technique. So we search for that instrument in other specialties. We go to the ophthalmology set and ENT set, we collect all these small sets, and we work on it. It is more challenging, but we proceed with that case and succeed.”

A third contributor pointed out the absence of modern technology, noting that surgeons had to rely primarily on their gained skills to navigate these limitations: “Absolutely, yes. That is one of the major issues in performing major reconstructive procedures there because of the unavailability of modern technology. I mean, endoscopic, endoscopic Laparoscopic, for example, laparoscopic instruments were not available, so we couldn't use any modern technology there. So most of the cases, we depended on our clinical sense to overcome this deficiency in modern technology.” Meanwhile, a fourth participant described challenging clinical situations where the quality of patient care suffered due to insufficient resources, sharing a harrowing account of a patient who died because of inadequate postoperative care and discussing the lack of critical diagnostics: “There was no good resources, for example, we had a patient who had a diaphragmatic hernia, but the ICU care was not the best. Unfortunately, the patient died at the end because postoperative care was not proper.”

These stories illustrate the dire situations that take place in LEDCs and the surgeons necessity to depend on their clinical judgement in the absence of adequate equipment. Another participant reinforced the universal challenge faced by volunteers due to equipment scarcity, emphasizing the need to prepare and bring vital supplies such as sutures and catheters themselves.

The sixth subject shared an incident highlighting the improvisation required when the electricity failed during an epispadias repair, with team members using phone flashlights to illuminate the surgical area and an anesthetist manually ventilating a patient for an extended period due to a ventilator outage: “And I remember many times that we operated on patients and the electricity goes off and the people around in the room, they have to put on their iPhone flashlight to continue the surgery. It happened in my room and in the pediatric surgery room and my room was ok because I was working on a patient with epispadias but in the other room that were working on a patient with major abdominal surgery, which was difficult to manage. It didn't happen only once, it happened several times. The electricity shortage is quite common and so the ventilation machine stopped, so we kept ventilating the patient manually.” This anecdote underscores the harsh realities and difficulties stemming from resource limitations in these environments.

### Infrastructure challenges

The topic of “Infrastructure Challenges” was the third most mentioned issue in our study, with 83 references pointing out the impacts of insufficient facilities, transportation difficulties, corruption, and the absence of basic services like water and electricity on the quality of surgical care.

One participant drew attention to the stark lack of advanced diagnostic tools and clean working conditions, detailing a specific instance where a key nuclear scan for diagnosing a urinary obstruction was not available, and the nearest facility offering such a service was 2,000 kilometers away. They painted a vivid picture of an overcrowded treatment room, bursting with patients and their families, while flies buzzed in a less than ideal setting.

Another subject recognized the inadequacies of the working environment but noted that efforts by local health institutions made the situation barely tolerable: The subject said, “...health institution there, they are playing very good effort to make the situation as optimum as they can and they make resources available for them as they can. And in general, it is an acceptable situation.” Despite a history of conflict affecting the infrastructure, the subject acknowledged the administrators' attempts to optimize what resources were available, deeming the overall situation “acceptable.”

A third individual lamented the unavailability of electronic or even proper medical records which hindered the effective tracking of patients' medical histories and physical examinations. Yet, they expressed a sentiment of adaptability, emphasizing the need to make do with the available infrastructure, including the sufficient number of operating rooms. They stated, “So the people are there are working with what is available. You have to adapt yourself to what is available. This is the available thing. So yeah, I mean it depends on what, what do you mean by infrastructure like do you mean there are there are rooms, OR rooms? Yes, there are like there are you know, yeah, I think that the message there is, is you have to work with what is available.”

In contrast, another participant pointed out inconsistencies in the quality of infrastructure, with some instances where finding an appropriate space for procedures was challenging, contrasting with times when everything was well-organized and ready from the outset.

The fifth participant highlighted how infrastructural barriers affected patients from lower socio-economic backgrounds who had to travel great distances for care. They shared, “those families, they can stay longer, far away of their families, they will come far away and they have to stay one maximum 2 days. And if you are in need to, them to stay longer, they have to pay for the hotel, which actually they, they don't have hotels there.” “Yeah, and also you will have some corruption. Also some people that come for 1 day, next day, the other people, they will come and they will say, ok, we are two actually, they are 1 and 1, you know, they, they are bored, they are asking for help anyway.” They also pointed out how organizational deficiencies could breed corruption, potentially allowing some individuals to manipulate the system for preferential treatment and causing delays in care for others.

Further emphasizing the issue of corruption, another volunteer noted how nepotism influenced patient prioritization, sometimes at the expense of those in dire need. They stated, “the ethical problems usually we faced is usually the nepotism, it is very common that to try to push patients who are related to them or related to the government officials.” This participant also mentioned the lack of essential infrastructure such as communication, electricity, water supply, and adequate building ventilation, recalling the sweltering heat during a particular time in Mukalla.

### Emotional and physiological challenges

The theme of “emotional and physiological challenges” came up frequently in our qualitative research, being mentioned 58 times. This theme helped us better understand the psychological stress experienced by surgeons and medical practitioners during their clinical duties.

For instance, Participant 1 described the physical toll of the job and the high volume of surgeries required, but also conveyed a sense of excitement and satisfaction. One statement from them was, “It definitely had some physical impact on me. It wasn't anything too severe to stop me, and I was actually eager to do it again.” They also spoke of the strain of long work hours: “We strive to see as many patients as we can, yet there's a schedule. Some days involved performing around nine or ten surgeries. We'd start in the morning and not finish until sunset.”

However, amidst these pressures, Participant 1 also expressed moments of emotional reward, feeling humbled and appreciative. They said, “It's quite humbling and makes you appreciate what you have. It drives you to be more resourceful and find creative solutions to problems.”

Participant 2 echoed the sentiment about the physical demands, describing the exhaustion after working long hours, but adjusting over time. Regarding the emotional rewards, they described a deep satisfaction from positively impacting patients and their families' lives: “You feel like you're giving new life to the patient and their family.”

Participant 3 reported no significant emotional or physiological difficulties, stating, “I didn't face any emotional challenges, and stress-wise I was fine.”

In contrast, Participant 4 spoke of profound emotional distress, which left a lasting impression. Despite this, they still felt a deep compulsion to help: “It was very emotional, especially in the early stages of my experience, and it left a scar on my soul. But, it's the nature of the work; we do what we have to do.”

Participant 5 shared no emotional struggles, instead focusing on the happiness derived from helping patients and receiving their gratitude.

Participant 6 recounted several stressful incidents, including confrontations with uncooperative staff during surgery, necessitating the intervention of hospital administration. They also highlighted the physical exhaustion from dealing with emergencies like power outages, requiring manual ventilation of a patient for an extended period.

Moreover, Participant 6 described the emotional weight of not being able to assist every patient, despite their exhaustive efforts. They said, “It's heartbreaking when you've helped many, but still, see a queue of patients waiting whom you know you can't help due to time constraints.” They also mentioned the physical and emotional implications of limited access to food, opting for fruit to sustain themselves during demanding work schedules.

### Effective communication with medical staff

The theme “Effective communication with medical staff” came up 42 times, indicating the unity among local doctors, healthcare workers, and visiting doctors who worked together as one team. One participant described how local doctors were often intimately involved with patient selection, which could create an emotional bond and a vested interest in their patients' improvement. They said, “Local doctors often joined in because they had chosen and connected with the patient personally.”

The same participant also noted the seamless teamwork between staff and emphasized that, despite dialectal challenges, communication within the team was still effective, especially since there were no issues understanding the medical terminology. “You'd think language barriers in certain parts of Yemen would hinder us, but we managed to understand each other just fine within the medical team,” they explained.

Another respondent praised the friendly and welcoming nature of the local doctors, attributing the smooth and efficient work environment to pre-existing relationships and respect. They added that, in their view, local health professionals were doing their utmost within a system that unfortunately didn't always provide adequate resources or support.

A third individual acknowledged communication was generally effective, though they did encounter some challenges when recruiting patients. Still, they were able to manage a substantial number during their visit.

The fourth participant highlighted the use of mobile phones for keeping in touch with local physicians when they were not present, ensuring continuity of patient care. They believed that the success of their mission relied heavily on this effective communication.

Another team member affirmed the significance of online communication tools like WhatsApp for ongoing contact after leaving the area. They spoke highly of the cooperation from local staff, experiencing no resistance or issues during their missions.

Yet another respondent spoke about the proactive nature of communication, starting well before missions commenced, sometime up to 2 months in advance with the creation of a WhatsApp group. They praised the local staff for their helpfulness and eagerness to learn, noting that while the Yemeni dialect was difficult, communication was still manageable and carried out with the assistance of helpful local staff.

The participant also discussed the importance of having local staff involved in explaining procedures and postoperative care to patients' families, ensuring that language barriers did not impede understanding. They emphasized the value of involving local colleagues in training and capacity building, seeing it as a crucial aspect of each visit.

Consistently effective communication—whether face-to-face, over the phone, or via online messaging platforms—was highlighted as central to the success of their collaborative efforts in providing healthcare. Despite language barriers and system limitations, the dedication and teamwork of both local and visiting staff shone through.

### Ineffective communication with medical staff

During visits to resource limited settings, surgeons frequently reported poor communication with local medical staff, noting this problem 36 times. Such communication breakdowns often led to ineffective teamwork with local staff and even disputes.

One surgeon recounted how requests for supplies during operations were met with delays, necessitating an abundance of patience. Despite efforts to improve the situation, the transient nature of their stays meant facing new challenges daily.

Another surgeon pointed out the local team's unfamiliarity with their procedures, necessitating teaching and reliance on the local staff's ability to learn quickly on the job.

A third surgeon lamented the lack of effective communication in some countries, which hindered case management.

Language barriers were repeatedly mentioned as a cause for concern, with one surgeon noting that their English instructions were often not understood, leading to medical errors. They shared an incident where this led to the misuse of a cautery machine.

Resistance to cooperation and learning from local physicians was another issue. One volunteer described having to take assertive action to maintain order and professionalism after recurrent instances of obstinacy.

Managing postoperative care presented another challenge. A surgeon mentioned that orders for patient care were often ignored, with prescribed medications not being administered. This negligence was attributed to a lack of attention and the unavailability of necessary medications.

Despite these hurdles, visiting medical professionals strive to adapt and overcome to provide the best possible care.

### Organizational and logistic difficulties

The section “organizational and logistic difficulties” cited 37 times, highlights the common issues faced with planning and organizing medical missions to resource limited settings. Problems such as delayed shipments, insufficient supplies, and inadequate facilities often compromise the quality of surgical care. For instance, one medical professional expressed surprise when crucial equipment failed to arrive on time.

Another recounted the struggles encountered with logistics, including using inappropriate sutures and oversized instruments for pediatric patients. These practitioners adapt as best they can, even when the sterility standards are suboptimal, focusing on preventing contamination.

The lack of both facilities and staff also poses major difficulties in managing patient volumes. One doctor described the overwhelming experience of providing consultations in corridors, due to the lack of available examination rooms, which speaks volumes about the difference in healthcare systems.

Despite meticulous planning, the sheer number of patients still extends doctors' working hours as they strive to treat as many as possible. Observations from a mission in Yemen described streets crowded with waiting patients, leading to sessions that could extend until late evening with some patients still hoping to be seen. Although the organization is typically adequate, the overwhelming case load means that many patients remain untreated.

Another significant hurdle is recruiting a sufficient number of patients, particularly when mission announcements come late. This limitation impacts the number of surgeries performed, even though the overall outcome of the mission may still be considered successful. Staffing issues persist, with recommendations to bring well-trained team members who can quickly instruct local staff to handle the exceptional demands of these missions.

### Lack of personnel

The section “Lack of Personnel” highlights the challenge of insufficient numbers of trained and suitable staff to support surgeons during medical procedures. There were 36 instances identified where due to a shortage of qualified personnel, doctors were forced to work alongside non-specialized nurses and hospital staff who had limited knowledge of the necessary surgical instruments. This deficiency contributed to prolonged surgery times.

Subject 1 shares their experience: “If you don't have a well-trained team, both surgically and in nursing, you're at a disadvantage. We had to use the staff available in the hospital. They weren't inept or inadequate; they simply lacked specialization, which was definitely problematic.”

They continue, “When you request an item during surgery, the response time is slow because the staff has to figure out what you need. Therefore, patience becomes a virtue. Despite efforts to prepare for the next day, similar issues would arise due to the brief duration of our stays. It meant facing new challenges daily.”

Subject 4 also comments on the difficulties, “There are times we're short of assistants, or we work with inexperienced ones, such as technician nurses, anesthesiologists, and assistant surgeons. This naturally leads to surgeries taking longer than usual. On occasion, we've even been unable to perform certain procedures because we lacked the specialist expertise on hand. For instance, we couldn't operate on burn patients needing scar release without a plastic surgeon.”

Regarding staff training, Subject 1 states that part of their mission was educating the local staff: “Training them was one of our key objectives.”

Subject 3 adds, “There were situations where staff members weren't familiar with the surgeries we were performing. We had to rely on educating them about our techniques and needs.”

The issue extended to anesthesiology, where there were not enough anesthesiology support, anesthesia machines, or know-how, creating obstacles for surgeries requiring general anesthesia. Subject 3 notes instances where pediatric surgeries couldn't be done: “We couldn't perform many pediatric surgeries under general anesthesia due to these limitations.”

Subject 4 recalls an incident in Malawi, “We encountered a situation where an anesthesia machine's tubing got blocked by fluids because it hadn't been used for a while, and the staff didn't know how to maintain it.”

Several subjects brought up the dire need for pediatric specialists. Subject 5 talks about the scarcity in Gambia: “In the whole country, despite having a population of over 3 million, there's only one recently graduated pediatric surgeon.”

Subject 6 reflects on the urgency in Yemen: “There isn't a single pediatric urologist in the entire country. Most are adult urologists who are not equipped to handle pediatric cases. Often, the information they provide us with is inaccurate.”

They lament that while some staff members had international training, the knowledge and practice level among local staff were at best rudimentary and at times perilously lacking.

### Personal risk and safety concerns

In the qualitative research design, the theme of “Personal risk and safety concerns” emerged frequently. Subject 1 briefly mentioned a major security issue. Subject 2 discussed safety concerns due to being in a war zone. Subject 6 delved into detailed accounts of security challenges posed by terrorist organizations, emphasizing the risks faced by both civilians and aid workers. They described the extreme safety measures taken, such as living in isolated areas, using heavily protected transportation, and being accompanied by a significant security detail. Additionally, Subject 6 noted the overlooked safety concerns related to food and sanitation, mentioning frequent instances of food poisoning among team members.

### Adapting to local disease patterns

The theme “adjusting to local health concerns” popped up 35 times in our discussions, highlighting the struggles visiting doctors face when they have to alter their surgical methods to deal with common conditions and the lack of resources. The first doctor we talked to talked about the need to get creative with generic tools, saying, “We sometimes had to make do with whatever instruments we had, even if they weren't the perfect fit for the surgery.” This improvisation also meant working with sutures and tools that they weren't used to. The second doctor pointed out that surgeries tended to take longer than back home due to the scarcity of proper surgical instruments, mentioning, “Sure, surgeries took a bit longer compared to in Saudi Arabia because we just didn't have the right tools for the job.” And these shortages meant that it was essential for them to bring key supplies themselves. The third doctor brought up that some surgeries had to be canceled because they just didn't have the necessary equipment, sadly noting, “Unfortunately, we had to skip some surgeries because we just didn't have the tools we needed.” The fourth doctor also highlighted difficulties, especially when dealing with children, as they had to use sutures and instruments that weren't suited for smaller patients, and they often had to rely on their expertise rather than advanced scans like ultrasounds or MRIs, which weren't available. “We usually had to go without any high-tech scans,” they said. The fifth doctor reinforced the importance of self-sufficiency due to the lack of preparation at the facilities: “You've got to have everything ready beforehand when you come.” Lastly, the sixth doctor emphasized how crucial training the local staff is, especially those working in anesthesia, to make sure surgeries are safe and effective, underscoring, “Training them is vital as well.” These comments give us a glimpse into how these visiting medical professionals had to adapt to the available resources and work on educating the local personnel to enhance the quality of their work, even in the face of resource constraints.

### Ethical dilemmas

The theme “ethical dilemmas” came up 31 times in our analysis, underscoring the complex reasons behind ethical challenges that visiting surgeons encounter while working in regions with scarce resources. Take, for example, the situation one practitioner faced in Rwanda, where a lack of diagnostic tools led to uncertainty about the best treatment option. They said, “We faced ethical issues where it wasn't clear what the best treatment was for the patients. Yet, we went ahead with the best option available at the time.” Another participant, working between culturally similar regions like Yemen and Saudi Arabia, didn't perceive any significant ethical issues, simply stating, “There is no difference.”

Contrastingly, a third contributor experienced fewer ethical dilemmas, attributing this to clear-cut cases and good organization: “Actually, most of the cases we handled were straightforward, so ethical issues didn't really come up.” Meanwhile, a fifth participant highlighted the ethical problem of corruption, indicating that some patients were unfairly favored: “Some people get help one day and others the next...but everyone is asking for help.”

Participant six shed light on a range of ethical challenges, such as nepotism and the dilemma of patient prioritization based on medical necessity vs. social connections. They recounted adapting surgical techniques owing to the lack of equipment while emphasizing the crucial consideration of patient safety, stating, “We frequently encounter ethical problems like nepotism... We often have to modify surgical techniques to ensure at least most of the procedure can be completed safely because we might lack specialist surgeons.”

These experiences from the field collectively illustrate the ethical quandaries that visiting medical personnel must navigate, encompassing issues of treatment decision-making in resource-limited settings, confronting corruption and nepotism, and maintaining ethical principles like fairness, doing good, and avoiding harm. The gathered accounts offer a window into the intricate ethical landscape inherent to surgical work in conflict-affected, resource-deprived areas.

### Complication rates

The “Complication rates” code was referenced nine times, focusing on the problems that emerged after surgery, as observed by the visiting surgeons. Subject 1 stated that the short-term outcomes were generally favorable, despite imperfect surgical environment conditions, such as saying, “In the short term, everything was going great.” They also noted the lack of hospital-acquired infections and wound complications, expressing surprise at the scarcity of anticipated complications, like stating, “You would imagine that hospital-acquired infections and wound infections would skyrocket, but no—we didn't have any of those.” Subject 2 underlined the heightened failure risk tied to surgical treatments in challenging environments, stating, “The risk of failure is higher.”

Subject 3 reported minimal short-term complications and no long-term complications due to regular interaction with the professional team in Gambia, sharing, “We did not have short-term complications...nothing mentioned about...long-term complications.” However, they confessed to the difficulties in the long-term follow-up process. Subject 4 described a case involving a patient's death due to insufficient postoperative care, emphasizing how resource limitations affect patient outcomes, “Unfortunately, the patient died at the end because the postoperative care was not proper.” They also acknowledged the challenge of evaluating long-term complications without proper follow-up, by asserting, “It's difficult to judge about the long-term complications.”

Subject 5 brought up communication limitations post-surgery, possibly hinting at potential challenges in monitoring and managing complications remotely, “After we leave, we have only contact by WhatsApp.” Subject 6 highlighted short-term complications seen during surgical procedures, attributing them either to the inherent risks of the processes or inadequate instrumentation, sharing, “Usually, we see more of a short-term complication than long-term complication.” They also underlined the need for multiple visits to the same facilities to address long-term complications that may arise, asserting, “So the second visit, some of those complications, if not all, presented itself to me again, to see it and to see, decide what to do with it next time.” This shows the different struggles and experiences faced by visiting surgeons in relation to postoperative complications in low resource areas.

### Skill and transfer training

Skill and transfer training is a key term aiming to ensure that doctors and staff effectively impart their expertise to local teams. This concept was referenced 13 times. Subject 1 observed the active engagement of local doctors, saying, “Local doctors would participate because they are the ones who interviewed and handpicked the patient beforehand,” suggesting a personal investment in patient care. They continued, acknowledging not only the dedication of local staff, but also their eagerness to learn: “The section head of the department... was a great guy overall and he was eager to learn new techniques that he could implement after we went home.”

Subject 2 expressed admiration for the local team's skills, stating, “The local urologist there... they are very intelligent, they have experience in the cases,” emphasizing their capability in handling case follow-ups.

Subject 3 initially faced communication challenges but found that “effective communication between us and the team there was the hallmark of success of that trip.” They underlined the efficiency in bridging the communication gap, ultimately leading to a successful mission.

Subject 5 addressed the necessity for specialized training in areas such as pediatric care, noting, “We have to teach them about the special procedures because they didn't have specialized care for the pediatrics.”

Subject 6 detailed their experience with local staff's willingness to learn, “There are very helpful people... and they were willing to learn.” They advocated for broadening the staff's education beyond medical skills to include professionalism and ethics: “One of the objectives of the campaign... that teaching them and training them how to become competent, not only as a physician, knowledge wise, but also in professionalism, in ethical [behavior], in communication.”

However, Subject 6 also revealed varying levels of success between two cities, Seiyun and Mukalla, in terms of skill transfer. “We're still working on training them in Mukalla... Seiyun is much well trained now.”

They emphasized the practice of involving local doctors in procedures to foster learning: “I always get the local doctor involved... so make them engaged in the decision making in the management approach in the surgical technique.” Yet, they also acknowledged that some local doctors might use their new skills primarily for private practice, which they accepted, as long as it was done “properly and I taught him really well.” This highlights the complexity of the impact of such training on local healthcare frameworks.

### Cultural and language barriers

The theme of “Cultural and Language Barriers,” referenced six times, encapsulates the communication challenges faced by the visiting medical teams in Yemen. These difficulties, stemming from language barriers and cultural differences, complicated interactions with both local staff and patients.

Subject 1 illuminated these challenges: “You would think that in Yemen everybody would be speaking Arabic... the dialect is quite difficult... you face that challenge where you try to understand sometimes what they are saying, not the medical staff because we didn't have that problem.”

Language barriers, as Subject 4 elaborated, can lead to gaps in patient history, impacting patient care: “We rely on the local physicians and surgeons... sometimes we don't have a good history because the language is a big barrier... the medical records in those countries is not the best. And sometimes we miss things in between which is not optimum.”

Subject 6 emphasized the specificity of the local dialect: “You have to understand... the dialogue is not really well understood by us... So it becomes really difficult to communicate with the patient.”

Even in the operating room, communication posed real risks. Subject 6 recounted a troubling incident where non-verbal cues were needed but also proved treacherous: “I usually speak in English... They don't understand my language... I have to point to them... Communication failure usually is an issue... one of them... put the cautery machine in the wrong way.”

To improve communication with patients' families, the visiting doctors relied on local healthcare professionals as interpreters, highlighting the crucial role of local partners. Subject 6 stressed the importance of this approach post-procedure: “I speak with the family... what we did, what we find... I used to have a local with me... a urologist from the same city because of the communication failure between me and the patient relatives.”

### Recommendations

The theme of “Recommendations” was cited in 24 instances, capturing the participants' advice for enhancing the efficacy of future medical missions and the preparation of contributing physicians. This section addresses numerous aspects of the mission, from team dynamics to the utilization of technology.

Subject 1 stressed the value of team cohesion: “You would go in these conditions and perform surgeries and I can't emphasize how important that you have a good team with you because it would alleviate much of that stress.” They also advocated for further development in telemedicine as a critical tool for patient recruitment: “Telemedicine played a key role in recruiting the participants and selecting the cases... It would play a much more important role if it was implemented further.” Additionally, Subject 1 called for meticulous patient selection and better preparatory information for the team: “Try as much as possible to preselect and filter the patients... know your limitations, both personal and environmental... assemble the best team that you can and be safe and do no harm.” They opined for additional resources to aid the campaign: “More resources, more resources, yeah, more resources, more people, more resources.”

Subject 3 underscored the significance of strong communication channels: “A major recommendation is optimal communication between the medical team here and the medical team in the local area... Knowing patients, knowing the history of patients prior to arrival... this will help us prepare...” They also noted the impact of telecommunication, even suggesting meeting patients through teleconsultations to bolster the mission's success.

Subject 4 highlighted that consistent visits build better relationships with local communities and improve service efficiency: “Maybe the best thing is to have consistency for one place... The best example is Tanzania where we used to go every 3 months.” They observed that although telemedicine, predominantly through phones, has been useful, real-time monitoring of vital signs could enhance care.

Subject 5 recommended more structured organization in patient preparation and specialized training for local staff. They acknowledged network challenges in Yemen affecting telemedicine: “If they have access for telemedicine there in Yemen... But if they have the telemedicine center there, we contact them, it can solve all the issues.”

Subject 6 advised thorough preparedness regarding instruments: “So prepare all instruments, have multiple settings for the instrument, prepare everything and visualize that you are going into the hospital.” Communication with local doctors was critical for them, especially regarding medication acquisitions. Additionally, Subject 6 gave personal advice to ensure one's health and safety: “Make sure that you don't over-eat. You don't eat anything... make sure about your safety, don't try to be a hero and do a blind visit without proper coverage or security.” They highlighted telemedicine's role in managing patient cases more conveniently: “Telemedicine can reduce the load on the clinic. I can see patients with nocturnal enuresis and I can manage them over the phone.”

## Discussion

As we begin our discussion, it is important to recognize that this qualitative phenomenological study delves into and focuses on the lived experiences of surgeons—specifically those specializing in pediatric urology—practicing within the constraints of resource-limited environments. Through comprehensive interviews, these professionals generously shared rich complex and interconnected data regarding their management strategies and adaptive approaches amidst the scarcity of resources often encountered in LEDCs. Several consistent trends were identified across these encounters. Among them, the challenges of inadequate equipment and infrastructure, a lack of medical personnel, and the extreme physical and emotional demands placed on the surgeons. These and other prominent trends will be explored in greater detail throughout the discussion. Presenting the resilience and ingenuity demonstrated by pediatric urologists as they navigate the complexities of delivering specialized care under less-than-ideal circumstances.

It is important to acknowledge the significant challenges faced by pediatric urologists in addressing the healthcare needs of patients from low socioeconomic backgrounds. These patients usually had incomplete medical histories and a limited understanding of health-related issues. Prior research, such as the study conducted by Olajide et al. showcased the detrimental impact of low socioeconomic status on healthcare outcomes, as individuals with limited resources may delay seeking medical attention until conditions worsen, potentially leading to more severe and chronic complications (11). Despite these substantial barriers, our study revealed that the support from the King Salman Humanitarian and Relief Center played a critical role in facilitating positive patient outcomes. The humanitarian missions were notably successful in part due to the commitment and compassion demonstrated by the visiting surgeons. Their dedication was not only related to their profession but also when it came to the service of those in dire need, with participating surgeons expressing profound personal gratification when witnessing the transformative effects of their work on both patients and their families. These surgeons often described their experiences as leaving a mark on their souls, indicating that the intrinsic motivation to provide care was a driving force in prevailing against the obstacles encountered during their missions.

A persistent theme throughout the interviews was the struggle to secure necessary resources and equipment for specialized procedures, a concern cited 85 times by participants. The gravity of this issue was highlighted by a surgeon's remark, “When you don't have the proper instrumentation to do a task, imagine a surgery; of course, things become more challenging, and surgeries take much longer.” Surgeons adapted to these constraints by using regular hospital rooms for both surgeries and consultations. Faced by several organizational and logistical obstacles, such as equipment shortages and delays in delivery, exacerbated by a disproportionate number of patients to available rooms. This often resulted in the use of makeshift tools and performing patient evaluations in hospital corridors. One critical problem reported was the intermittent loss of electricity during significant surgical procedures, necessitating manual ventilation until power was restored. These accounts emphasize the need for visiting medical professionals to be adaptable, ready to confront unexpected challenges with resolve and ingenuity. Consistent with findings from other research, such as the study by Lelli Chiesa et al. resource deficits in hospitals were evident, including the absence of critical departments and equipment like pathology labs, isolated treatment rooms, infusion sets, flow meters, and filters (10). Echoing our findings, Tim et al. contended that despite these resource gaps, the most crucial determinant of surgical outcomes remained the surgeon's expertise (9).

The shortage of qualified personnel posed a challenge for surgeons in this setting. Operating rooms were often understaffed, leading to surgeons having to perform surgeries simultaneously in neighboring rooms. In Gambia, an entire nation relied on just one recently graduated pediatric surgeon. This scarcity was accentuated in a study by Lelli Chiesa et al., who noted that in Africa, there was merely one pediatric surgeon for nearly every six million children (10). Moreover, the lack of qualified local nurses further complicated matters, with 15 out of 45 nurses being recent graduates lacking adequate training. Similar challenges were echoed in a study by Tim et al., emphasizing how the absence of proficient local surgeons significantly impacted surgical outcomes. Security risks added a critical layer of complexity to their work environment, with safety concerns paramount (9). Personal safety became a primary concern given the precarious situations encountered. This necessitated high-security support from the King Salman Relief Center due to constant threats from local terrorist groups and the presence of improvised explosive devices (IEDs). As a result, surgeons often found themselves in high-stress environments with compromised communication. An incident highlighting the need for swift action occurred when hospital management had to intervene to address disrespectful behavior, underscoring the challenging dynamics within the team.

Another important theme which arose: the emotional and physical toll on surgeons providing care in these difficult environments. Participants frequently cited long working hours under challenging conditions—often compounded by security concerns—as sources of significant emotional distress. Narratives revealed that most surgeons encountered some degree of psychological strain, a finding substantiated by no fewer than 58 mentions throughout the interviews. Among the factors contributing to this distress, physical fatigue and the relentless pace of surgical workload featured prominently. Surgeons shared personal accounts of their grueling schedules, with one stating, “Yes, physically, I was exhausted. Yes, we spent from seven or 8 a.m. till 6 p.m.” Despite acknowledging the hardship, the dedication to providing care remained unwavering. Another surgeon reflected, “While we operated daily, it did take a toll on anybody, but nothing too extreme. It had some physical impact, but it wasn't major enough to deter me.” These insights underscore the complex reality of healthcare providers operating in resource-limited settings—balancing the demands of delivering urgent care while grappling with their own human vulnerabilities.

Effective communication with the local staff emerged as a critical factor in ensuring the smooth operation of the missions. The local team's role was pivotal, as evidenced by their assistance with patient preparation and follow-up care with a comprehensive postoperative plan, even after the visiting surgeons had departed. One participant emphasized, “The key to our trip's success was the quality of communication between our team and the local staff.” Furthermore, this collaboration facilitated the visiting doctors' interactions with the local community.

Notably, the significance of effective communication is mentioned in the literature as well. Geiling et al. research advocates for the importance of communication in enhancing the success of medical missions, particularly in resource-limited areas. They argue that any breakdown in information exchange can result in care that is both inefficient and ineffective (2).

### Optimizing international collaborations in pediatric urology: insights and implications

In our study, “From Crisis to Care: Exploring the Resilience of Pediatric Urologists in Tackling Complex Urological Challenges in a Resource-Limited Country During Volunteer Campaigns: A Qualitative Study,” participants provided valuable recommendations for enhancing the efficacy of future missions and campaigns. They emphasized the importance of assembling a strong, cohesive team to significantly reduce stress. Effective communication between visiting and local medical teams is vital when it comes to the planning and care of patients prior to the surgeons' arrival, along with meticulous preparations for each mission.

The benefits of establishing and maintaining a consistent presence in given locations were highlighted due to the incremental improvements in handling staff dynamics, language barriers, infrastructure complexities, and equipment issues. Through repeated missions, surgeons become familiar with local challenges, enabling them to navigate and address the nuisances more effectively.

Empowering the local medical staff through education was cited as crucial for providing specialized and sustainable care. Furthermore, the study found that telemedicine is an underrepresented resource in resource limited settings, which has the potential to alleviate clinic strain and enhance long-term patient management, follow-up care, and overall patient satisfaction.

Our paper encountered several limitations that may have influenced our interpretation and caused bias. First the audio quality during interviews which could be attributed to poor internet connection and varying microphone quality. These technical issues occasionally made it difficult to hear the speaker clearly. This impaired our ability to fully understand and capture responses. Even though a transcription software was used and a rigorous manual review process to overcome this challenge, there remains the possibility that emotional cues, subtle details, and nuances may have been overlooked. Overall this may have affected the accuracy of the data and consequently our results. Furthermore these audio issues may have caused us to rely on more clear and audible responses potentially skewing the representation of data.

Second another limitation was the lack of geographical diversity in our study sample, with the majority of surgical experiences being reported by subjects who worked in Yemen. This regional bias poses an issue as the perspectives and challenges may not translate to experiences in other resource limited settings. The surgical experiences described may have been shaped by the unique sociopolitical environment of Yemen. This limitation poses the issue of the generalizability of our findings. Future research should aim to include a broader range of resource-limited countries to capture a more diverse array of experiences and perspectives.

Third a limitation that arose was the reliance on self-reported data which risks the possibility of both social desirability and recall bias. Subjects may have misremembered details or presented them in a more favorable light. This could have been an issue especially when discussing sensitive topics. This could have influenced the presentation of information either being overly positive or incomplete portrayal.

These limitations highlight the need for further research where limitations are optimized. This would enhance generalizability and provide more comprehensive understandings of findings. We also recommend the usage of triangulation data collection methods in order to validate self-reported data and decrease the influence of recall bias. Acknowledging these biases strengthens our interpretation of the data and highlights the need to be cautious when applying the data to other settings.

## Conclusion

In conclusion, this study highlights the multifaceted nature of pediatric urology in resource-limited settings, where practitioners navigate through a landscape marked by equipment shortages, inadequate infrastructure, and a lack of skilled personnel. These bottlenecks not only affect surgical outcomes and patient safety but also lead to stress among healthcare providers. Despite these hardships, surgeons display remarkable resilience, employing coping strategies ranging from personal motivation to community support, often buoyed by the critical backing of the King Salman Humanitarian and Relief Center. Acknowledging these challenges, the psychological impact, and the adaptive responses is crucial. It underlines the importance of targeted interventions to improve care quality and the health and welfare of both patients and healthcare professionals in challenging environments.

## Data Availability

The raw data supporting the conclusions of this article will be made available by the authors, without undue reservation.
