# Supplementary material for: From crisis to care: exploring the resilience of pediatric urologists in tackling complex urological challenges in a resource-limited country during volunteer campaigns: a qualitative study
Source: Front Public Health. 2025 Apr 17;13:1486283. doi: 10.3389/fpubh.2025.1486283 (PMC12043668; doi:10.3389/fpubh.2025.1486283)
Supplement: Supplementary file 1 [file Data_Sheet_1.pdf]

Supplemental information :

The interview questions were carefully crafted to elicit valuable insights and were structured as follows:

1. Can you tell me a little about your professional background.
2. How many times have you had trips to these resource-limited settings and what motivates you to provide care in these areas?
3. can you describe the communication and collaboration with other healthcare professionals involved in the care of these patients? How effective was your communication?
4. Have you encountered any ethical or cultural considerations specific to the management of these conditions in resource-limited settings? How have you addressed them?
5. Due to a lack of access to modern technology, what were the alternatives when it comes to collecting patient data and past (recorded) history for optimal management of the patient
6. Can you describe the major challenges you have encountered while managing pediatric patients with complex urological conditions in resourcelimited settings? How have you adapted your surgical techniques or approaches to overcome these challenges?
7. how do the newly adapted techniques affect the length of the surgery?
8. How do you coordinate postoperative care and follow-up for these patients and their families in resource-limited settings?
9. Have you encountered significant obstacles in terms of infrastructure, equipment, or staffing in resource-limited settings? How have you worked around these obstacles?
10. Have you encountered a difficult situation where you had to depend on your own clinical judgment rather than requesting the needed investigation in resource-limited settings, and how do you think this affected the outcome?
11. Can you share any personal experiences where you faced significant emotional challenges while practicing pediatric surgery in a limited-resource setting, and how you overcame them?
12. What are some prevalent short and long term complications you have faced due to the improvisation of certain surgical techniques were scarce and how were they managed?

13. What obstacles do you think patients face when it comes to post-surgical recovery?
14. What are the long-term outcomes you have observed in pediatric patients with complex urological conditions in resource-limited settings, and how do they compare to patients in resource-rich settings?
15. was the healthcare team able to ensure access to these specialized surgical procedures for the numerous patients each with different limitations in seeking this specialized care
16. Based on your experiences, what recommendations do you have for optimizing surgical management and improving long-term outcomes for these patients in resource-limited settings?
17. Based on your knowledge and experience, what do you think are some necessary steps that should be taken in order to improve healthcare provided in these resource-limited areas?
18. How do you perceive the role of telemedicine or teleconsultations in improving access to specialized care for pediatric patients with complex urological conditions in resource-limited
